# Low-dose capecitabine (Xeloda) for treatment for gastrointestinal cancer

**DOI:** 10.1007/s12032-014-0870-2

**Published:** 2014-02-08

**Authors:** Jasmine Miger, Annika Holmqvist, Xiao-Feng Sun, Maria Albertsson

**Affiliations:** Division of Oncology, Department of Clinical and Experimental Medicine, Faculty of Health Sciences, County Council of Östergötland, University of Linköping, 581 85 Linköping, Sweden

**Keywords:** Capecitabine, Gastrointestinal cancer, Metronomic treatment, Response, QoL

## Abstract

The prodrug capecitabine (Xeloda) has been an important drug for treatment for gastrointestinal cancer (GI-cancer). This study explores the efficacy of continuous metronomic Xeloda, as well as tolerability and best response during treatment. Patients (*n* = 35) with stage IV GI-cancer were included in the study and were divided into two groups; upper (*n* = 13) and lower (*n* = 22) GI-cancer. All patients were given continuous metronomic Xeloda (500 mg × 2). Best response was measured by radiological and clinical examination including laboratory results. Standard RECIST criteria were used. Median age was 66 (range 29–86). Those patients who received first and second line had the longest duration of treatment. For patients with metastatic gastrointestinal cancer, metronomic capecitabine (Xeloda) may be beneficial both as far as tumor control and quality of life is concerned. In this pilot study, palliation for more than 2 years is observed for 6 of the 35 patients.

## Introduction

5-Fluorouracil (5-FU) has been the backbone of treatment for gastrointestinal cancer (GI-cancer) since the discovery of its efficacy in the fifties. In spite of the wide experience in clinical oncology, there are still controversies regarding the optimal administration of the drug. The latest development with an oral administration of the prodrug capecitabine opened new possibilities in this matter [1]. In this pilot study of patients with tumors in the GI tract, the efficacy of continuous metronomic capecitabine was explored. We investigated both the tolerability and the best response to treatment.

Another goal with the study was to investigate whether the drug was efficient in an elderly patient population given in a continuously low-dose setting.

This was the only alternative to treat the patients since the normal standard chemotherapy regimen was considered too toxic. Here, patients with severe side effects from conventional treatment were included.

## Materials and methods

### Study design and patients

This pilot study included 35 patients in stage IV (GI-cancer) with primary metastatic or recurrent disease planned for treatment during 2010–2012. Patients were recruited from one county in Sweden. Seventeen of the 35 patients were more than 70 years of age, ranging from 29 to 86 years.

Many of the patients were pretreated with conventional chemotherapy before treatment with low-dose capecitabine.

The group treated with metronomic capecitabine in the first line was patients who were not considered suitable for conventional treatment mostly because of high age or medical contraindications. The group with this treatment in second line was generally patients with severe side effects after conventional chemotherapy.

**Fig. 1:**
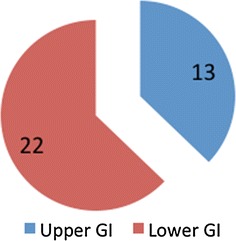
The number of patients in the two groups with upper and lower GI-cancer

We divided the patients into two groups as shown in Fig. 1; one group with upper GI-cancer and one with lower GI-cancer.

The malignancies representing the upper GI-cancer were carcinoma of the esophagus, the hepatobiliary system and the pancreas. Malignancies representing the lower GI-cancer were carcinoma of the colon and rectum.

The treatment with low-dose capecitabine (500 mg × 2) was considered for patient with a high age who received toxicity grade III–IV to prior chemotherapy or because evidence-based alternatives had already been tested.

The patients were followed every 4th week with clinical examination and laboratory measurements in an outpatient basis. Radiological examination with response evaluation was done approximately after 2 months of treatment, and RECIST criteria were used.

The patient data included the information of recurrent disease, start of treatment with low-dose capecitabine, the reason for treatment as well as duration of treatment. Other anticancer drugs during study were noted and also other malignancies present.

Since the follow-up was quite frequent, the best response during the treatment for most patients could be easily determined. End points used in this study were progress, regress or stable disease as well as the cases where no assessment of response was possible.

## Results

Since the material is heterogeneous, we wish to illustrate them by describing three individual cases as following.

The first patient received treatment with capecitabine and bevacizumab as the fifth line of treatment (Nr 28) (see Table 1) was a women born in 1973. She was diagnosed with cecal cancer in 2003. There was a doctor delay of more than a year, and when she finally was diagnosed, she had metastatic disease. She lived with metastatic disease for 9.5 years with intermittent chemotherapy and most of the time with good quality of life (QoL). Shortly, before her death, she participated in a horse show competition. She received metronomic capecitabine and bevacizumab for 2 years.

**Table 1:** Patient data

Case	Date of diagnosis	Months until recurrence from date of operation	Year of recurrence	Start of treatment	Days of survival from date of diagnosis	Date of death
1	April 2009		2010	January 2012	1,138	May 2012
2	October 2007	29	2010	December 2011	1,635	April 2012
3	August 2010	4	2011	December 2011		
4	August 2009	24	2011	January 2012		
5	November 2008	16	2010	January 2011		
6	November 2010	10	2011	March 2012		
7	November 2011			March 2012		
8	March 2009			August 2009	447	May 2010
9	October 2009			December 2011	912	April 2012
10	May 2010			September 2011	400	June 2011
11	August 2010	5	2011	December 2011		
12	August 2009	?	2011		812	November 2011
13	December 2006	49	2011	May 2011	1,736	September 2011
14	April 2006	24	2008	April 2011	2,223	May 2012
15				October 2010		
16	September 2010			January 2011		
17	December 2007	48	2012	August 2011		
18	July 2007	4	2008	July 2011	1,552	October 2011
19	August 2004	76	2011	September 2011		
20	September 2010			January 2011		
21	June 2011			September 2011		
22	October 2010			August 2011	379	November 2011
23	December 2005	56	2010	January 2011	2,181	November 2011
24	October 2008	14	2010	April 2011		
25	October 2011			October 2011	68	December 2011
26	March 2007	10	2008	August 2010	1,757	January 2012
27	March 2007	34	2010	September 2010	1,512	April 2011
28	July 2003			August 2009	3,109	January 2012
29	October 2004	36	2007	November 2011	2,740	April 2012
30	April 2012			May 2012		
31	June 2010			April 2012		
32	September 2011			March 2012		
33	August 2010	3	2011	May 2012		
34	January 2011	5	2011	June 2012		
35	March 2011			November 2011		

The second patient (NR 20) (Table 1) is a younger man born in 1977. He was diagnosed in 2010 with cancer of the hepatobiliary system and lung metastases. He started with conventional chemotherapy with severe side effects after two cycles. He was motivated for treatment but not physically fit which was the incitement to switch to metronomic capecitabine. A partial regression (PR) was observed. Radiofrequency (RF) treatment was given to a complete response in the liver in October 2012. He has shown stable disease by now for more than 2 years in the lungs being able to work full time and is alive and well.

The third patient receiving metronomic capecitabine (NR 21) (see Table 1) is a man with adenocarcinoma diagnosed with a liver biopsy at the age of 86 years. When diagnosed, the multidisciplinary team conference hesitated to give him treatment. While still motivated for treatment, he made sure to be accepted for doctors’ appointment at the oncology department. Treatment was started with metronomic capecitabine and was well tolerated without any side effects. Three months later, radiology confirmed regress of the disease, however, not a PR. He had stable disease for 1 year. Because of progress, he is now without tumor-specific treatment and has lived 2 years with good QoL and no side effects of treatment. Since his response to the treatment was good, he was reconsidered for local treatment. Because of the location of the tumor nearby big vessels, RF treatment was not an alternative. He was offered stereotactic treatment but declined when he was informed of possible side effects. He is alive and well.

Within the group selected for this treatment because of unacceptable toxicity grade III–IV (13 patients), only 3 of them were 75 years or older.

In the group with upper GI-cancer, about 30 % (*n* = 4) showed regress of disease (PR), 23 % (*n* = 3) progress and 46 % (*n* = 6) stable disease. In the group with lower GI-cancer, about 61 % (*n* = 11) showed progress and 39 % (*n* = 7) stable disease.

**Fig. 2:**
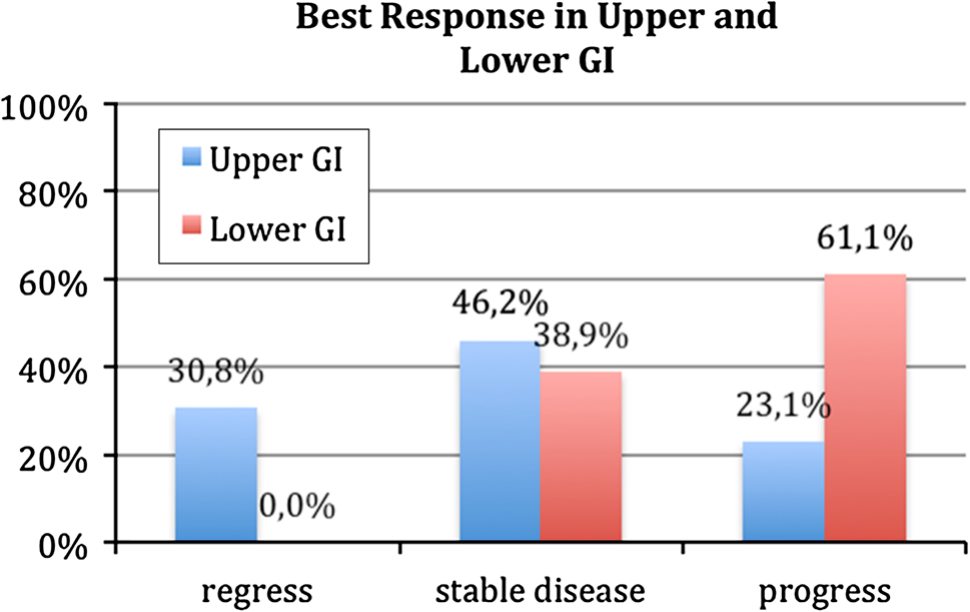
Best response in both groups

**Fig. 3:**
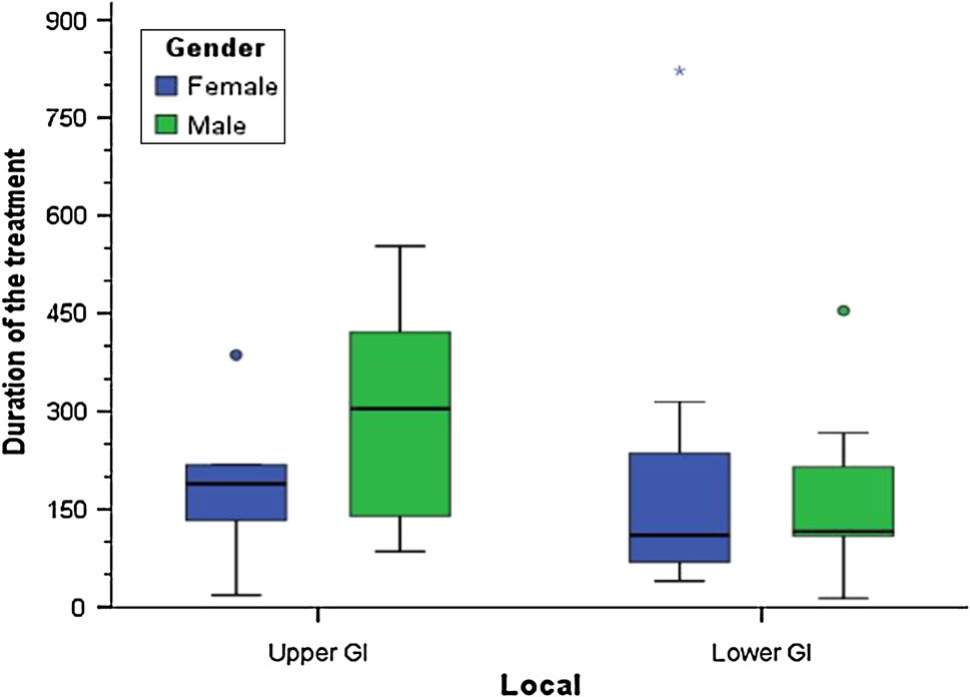
Duration of treatment

Figure 2 shows best response divided between men and women, and Fig. 3 shows the duration of treatment for both men and women in the two groups. Obviously, treatment duration was longer for upper GI-cancer. Still, the median duration of treatment was quite long for both groups. As shown in Fig. 3, one patient with the duration of treatment of 822 days was the young woman described above (NR 28). The other two dots present other two patients with an exceptional long duration of treatment.

## Discussion

The number of people diagnosed with cancer is increasing in Sweden. One of the reasons is thought to be an increasing healthy elderly population.

A woman of 65 years old has a life expectancy of 20 years more and man about 18 years. About 60 % of the patients diagnosed with cancer are 65 year or older. Still, they are rarely included in research studies regarding new methods or treatments. Studies from USA as well as Sweden show that there are a great number of older patients not receiving any antitumor treatment at all [2]. The reason is not yet established but might be the lack of information of proven therapies as well as resignation.

Colorectal cancer is the third most common diagnosis among both men and women in Sweden. There are approximately 5,000 new cases of colorectal cancer diagnosed every year. Almost half of those are over 75 years old.

The incidence increases with increasing age and doubles every seventh years for patients over 50 years of age. There is a lack of information about the optimal treatment regarding efficacy as well as QoL in patients suffering from CRC over 70 years of age, who often suffer from other diseases as well. Within the palliative setting, it is important to try to prevent disease progression without compromising patients QoL more than necessary. A continuous low-dose approach is not commonly used for patients with gastrointestinal cancer; however, earlier reports have been done both for esophageal cancer, breast cancer and prostatic cancer [3]. The patients within this report were those with very severe side effects with conventional treatment and patients with an old age where the clinician hesitated to offer conventional toxic treatment. Moreover, a group of patients were palliated during a long period where the conventional treatment regimes all have been explored.

Of these 35 patients, six are still alive more than 24 months after initiation of treatment, all with active disease but stable disease and good QoL. Of these six, two had mucous adenocarcinoma, which we know is often therapy resistant, three patients had cholangiocarcinoma and one had adenocarcinoma in the liver.

Age is measured chronologically in many circumstances. From a medical perspective, the chronological age is more irrelevant as the patient becomes older. In a group of 80 years old, you can find healthy physically active people, and persons with many diseases who require a lot of care and help. Differences in treatment can be motivated but sometimes motivations for the differences are not fully explained other than the chronological age of the patient.

With the help of magnetic resonance imaging (MRI) and computed tomography (CT), we can study the affected organ/organs with accuracy. The disadvantage of this is also that we receive more information than we would like, as we can notice other pathologies not symptomatic for the patient. Since this is seen especially in older patients, examination with MRI or CT is excluded in many cases even though metastases will not be found. This is not acceptable, and neither is the fact that many older patients are excluded from multidisciplinary conferences.

The relative 5-year survival for all cancer diagnoses today is 67%, and 36 % in the beginning of the 70s indicating that the prognosis for cancer patients has improved tremendously [4].

It is important to give optimal treatment to patients with colorectal cancer. However, there is a lack of information on treatment efficiency and safety for CRC in the oldest to old patients often suffering from underlying comorbidity. Lack of precise knowledge may also influence an option of providing a palliative care only [5, 6].

Our study shows that for the patient with toxicity to conventional chemotherapy or old age, metronomic capecitabine (Xeloda) can serve as an alternative treatment. Both tumor regression and SD were observed.

This study could be the incitement for a future randomized prospective study among the elderly patient group. A proper patient group would be patients with an age of 75 years or older with metastatic CRC disease, an ECOG performance status of 0–1 with acceptable hematological, liver and renal function tests. Results from such a study will fill a huge knowledge gap benefitting the emerging elderly population with CRC.
